# A pathologic mandibular fracture revealing a bifocal location of Langerhans cell histiocytosis

**DOI:** 10.1016/j.amsu.2020.06.019

**Published:** 2020-06-26

**Authors:** Maamouri Sabrine, Ben Rejeb Marouen, Ines Riahi, Zitouni Karima, Zanidi Nadia, Zairi Issam

**Affiliations:** a Department of Maxillo Facial and Aesthetic Surgery of Charles Nicole's Hospital, Tunis, Tunisia; b Department of ORL of Charles Nicole's Hospital, Tunis, Tunisia; c Department of Anatomopathology of Charles Nicole's Hospital, Tunis, Tunisia

**Keywords:** Pathologic fracture, Mandibular location, Histiocytosis

## Abstract

**Introduction:**

Langerhans cell histiocytosis is a rare disease. When it occurs in the cranium/facial bones, the mandibular location is the most frequent.

**Presentation of case:**

A 31 years-old man was referred to our department for a mandibular chronic discomfort during an acute exacerbation, **spontaneous teeth mobility and an alteration of the dental occlusion revealing** a pathologic mandibular fracture.

The diagnosis was confirmed by a subsequent CT scan.

**The surgical procedure was performed under general anesthesia by a maxillo-facial senior surgeon.**

**The therapeutic plan combined teeth extractions, enucleation of both the left maxillary and right mandibular cystic lesions and osteosynthesis of the pathologic mandibular fracture with a miniplate.**

Histological and immunohistochemistry analysis of the maxillary and mandibular cystic lesions **pointed the diagnosis of a bifocal** Langerhans cell histiocytosis of the oral cavity.

**Several investigations were done in order to find another location, showing no abnormalities.**

**Discussion:**

This is a case of rare single system LCH at two distinct locations: one at the mandibular bone and the other at the upper left maxilla. Both were uncovered by an acute exacerbation of a chronic discomfort secondary to a mandibular pathologic fracture. This should draw attention to the issues of the diagnosis.

**Conclusion:**

An early LCH diagnosis and a multidisciplinary treatment plan allow the improvement of the patient ‘s prognosis and quality of life.

## Highlights

• LCH is sub classified according to site of manifestation and organ involvement.
• Facial locations are rare, and mandibular involvement is the most frequent among them.
• Investigate unusual findings such as a third-degreeteeth mobility in a young patient with no history of periodontitis.
• Histological proof is a key tool.
• Treatment depends on the number and locations of the lesions.

## 1 Introduction

Langerhans cell histiocytosis (LCH) is the latest terminology for a disorder of the reticulo-endothelial system [1]. The etiology of the disease is still unknown, and there has been considerable debate whether LCH represents an inflammatory or a neoplastic disease [2,3].

Also, the clinical spectrum of this disease varies from a simple eosinophilic granuloma to a multi-organ involvement.

Treatment options vary depending on the extent of the disease and the severity at discovery.

Recently the Histiocyte Society has published a revised classification of histiocytosis in which LCH is sub classified according to the site of manifestation and organ involvement: single system LCH, lung LCH and multi system LCH with or without risk organ involvement (risk organs: liver, spleen, bone marrow) [4]

Facial locations are rare, and mandibular involvement is considered as the most frequent among them, occurring mostly in young people aged less than 20 years [1]

Here we display a patient presenting an LCH case with both maxillary and jaw location revealed by a mandibular pathologic fracture. The aim of this paper is to ease early recognition of this potentially aggressive disease.

**This work has been reported in line with the SCARE criteria.** [5]

## 2 Case report

A 31 years-old man was referred to **our department of maxillo-facial and aesthetic surgery in Charles Nicole's hospital** for a mandibular chronic discomfort with an acute exacerbation revealing a pathologic mandibular fracture.

**Our patient characteristics and past medical history did not reveal any abnormalities:**

**No relevant genetic information, family history, chronic diseases, medication, and no smoking history.**

**Also, environmental factors including lifestyle and psychosocial background for the risk of degenerative diseases were investigated revealing no remarkable features.**

Extraoral examination showed a left lower face swelling and tenderness without hypoesthesia nor cervical lymph nodes.

Intraoral examination revealed poor oral hygiene, and impairment of the periodontal support with third degree mobility of teeth belonging to second, third and fourth quadrant.

**Fig. 1:**
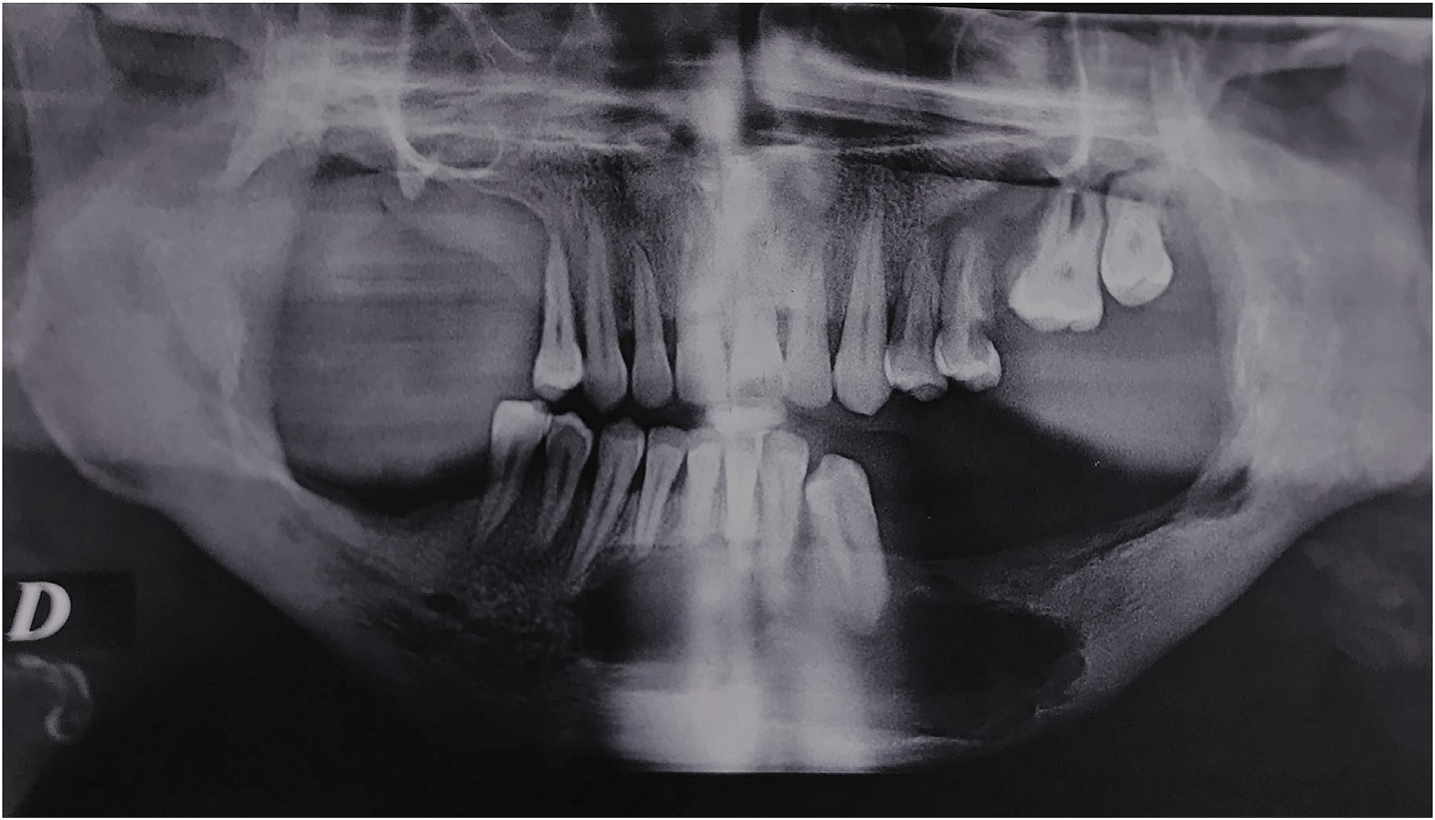
Pre-operative orthopantomogram showing bifocal cystic lesions (mandible and left maxilla) and multiple radiolucent areas in the alveolar ridge.

The orthopantomogram showed multiple radiolucent areas with well outlined borders on the mandible and on the left maxilla with severe alveolar bone resorption (Fig. 1).

**Fig. 2:**
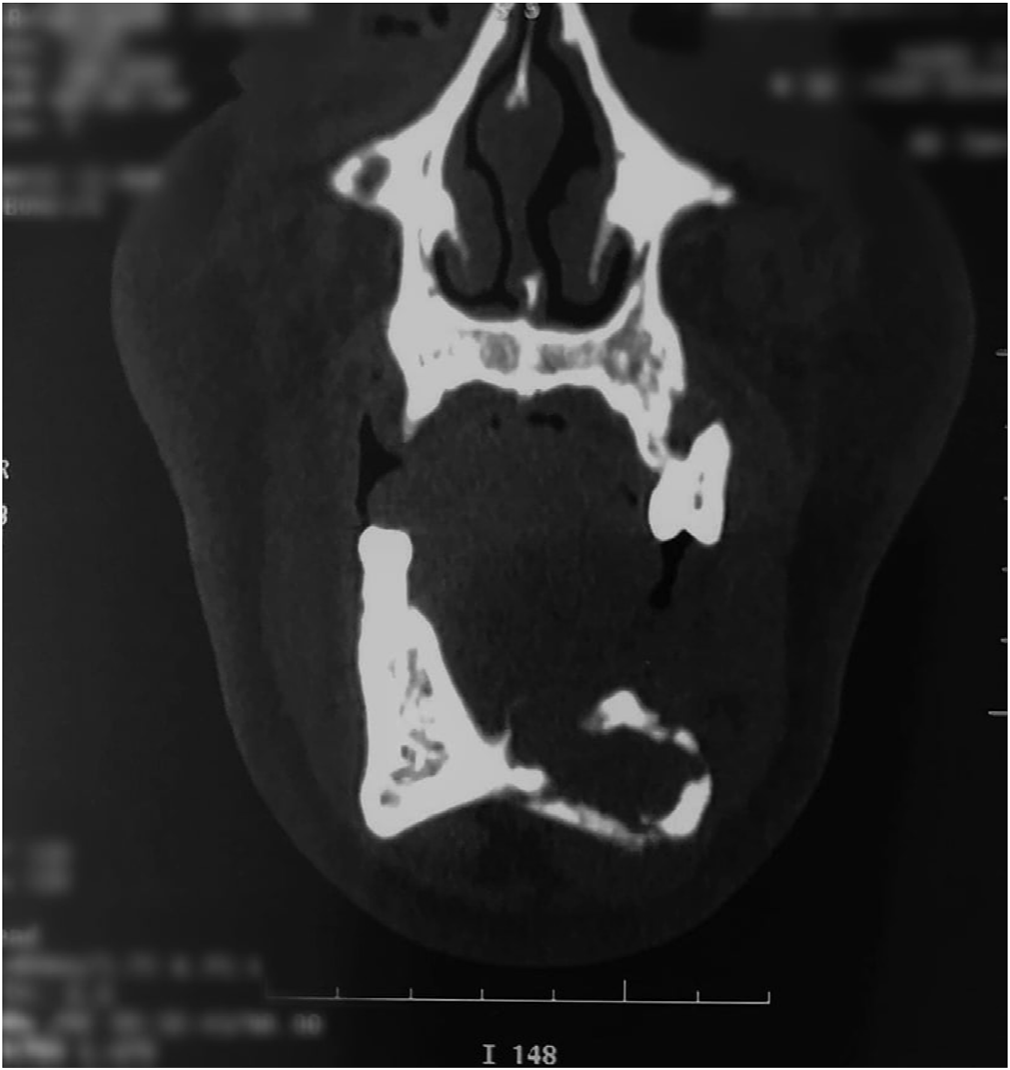
CT scan with frontal section showing an osteolytic mandibular lesion with a maxillary floating tooth.

**Fig. 3:**
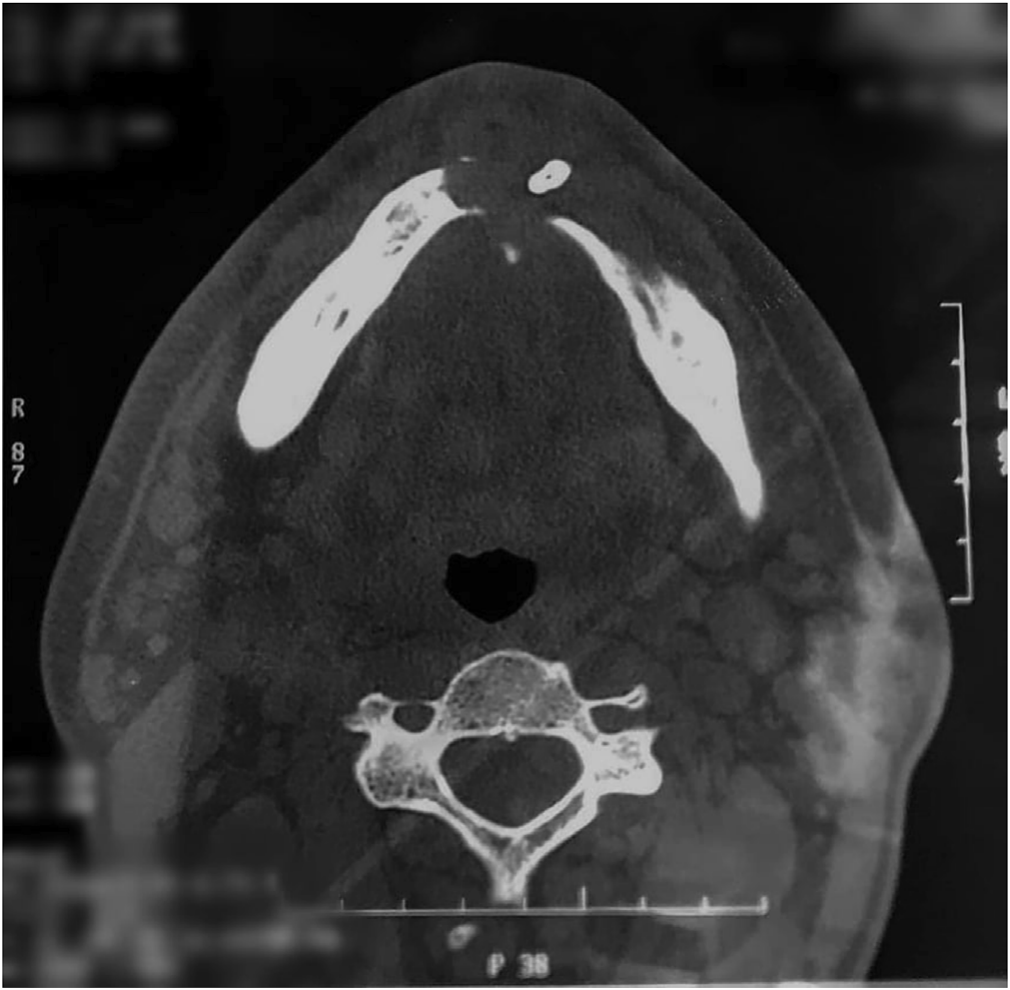
CT scan with an axial section showing the mandibular cortical interruption secondary to an osteolytic lesion.

**Fig. 4:**
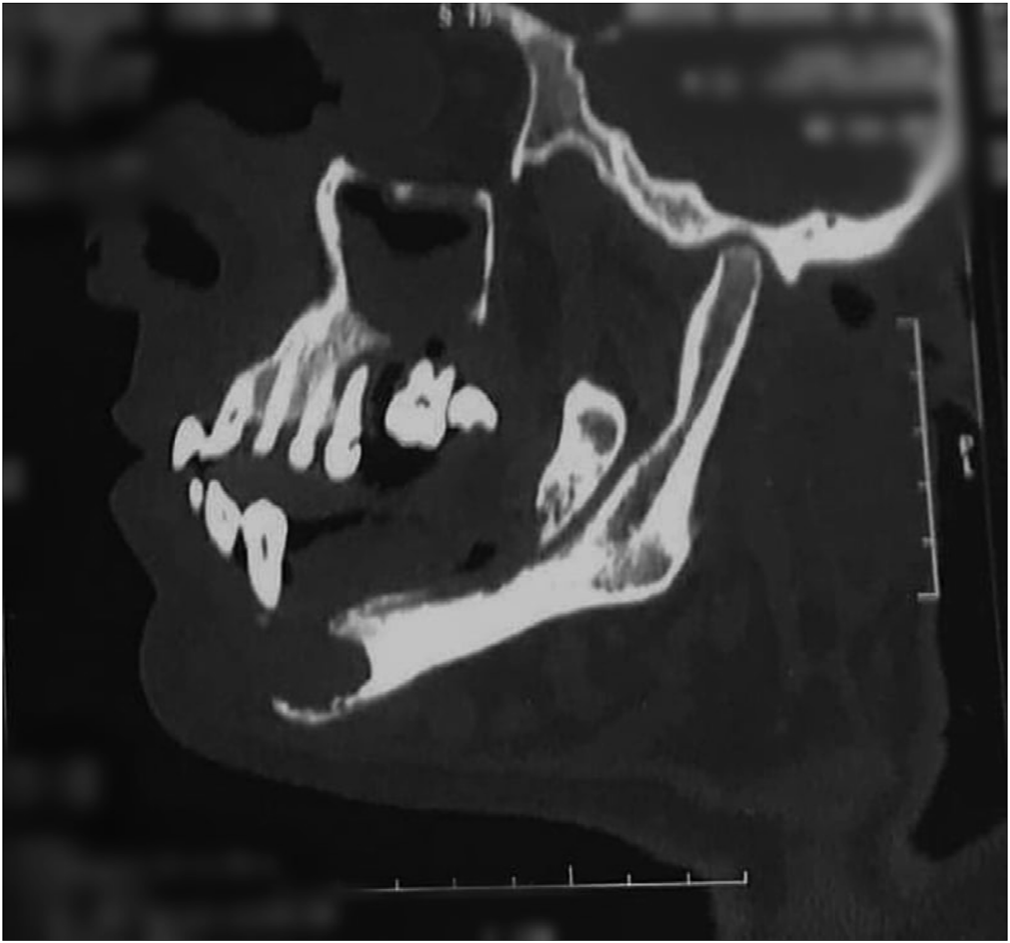
CT scan with a sagittal section showing bifocal (maxillary and mandibular) osteolytic lesions.

**Fig. 5:**
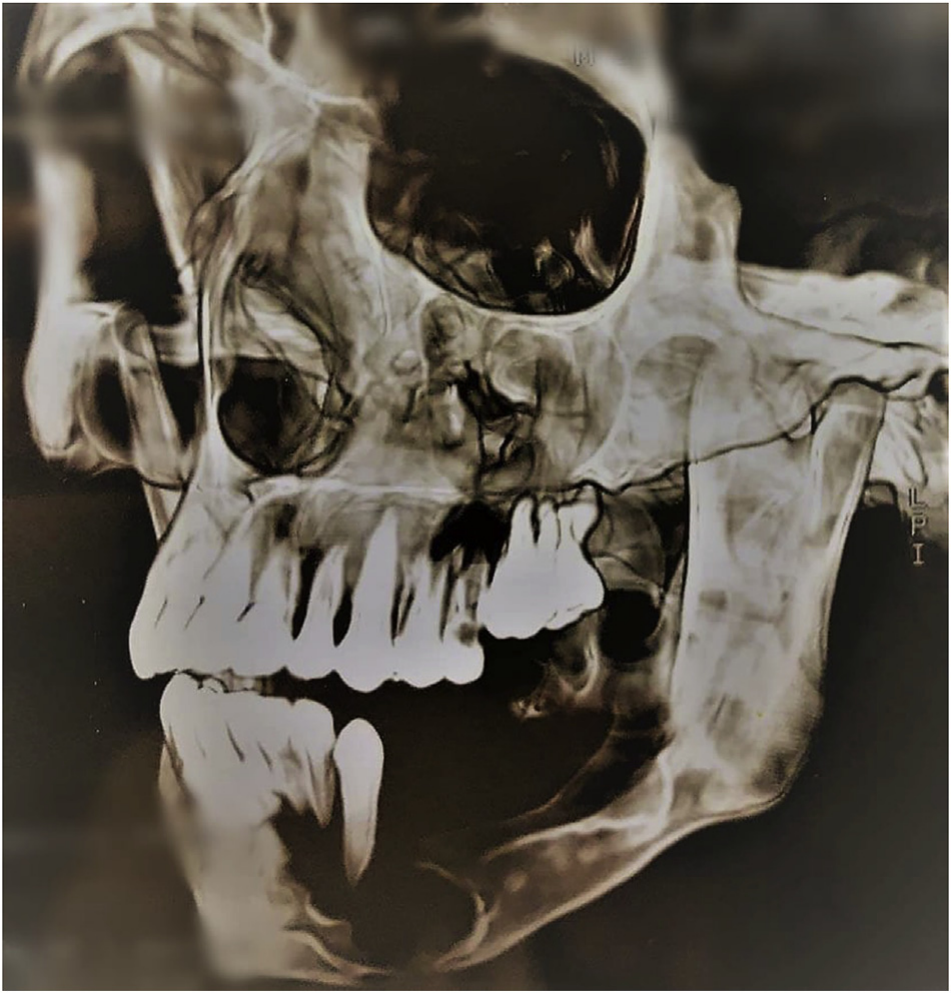
CT scan with 3D reconstruction :3/4 incidence showing multiple radiolucent areas with well outlined borders on the mandible with a fracture and on the maxilla.

**Fig. 6:**
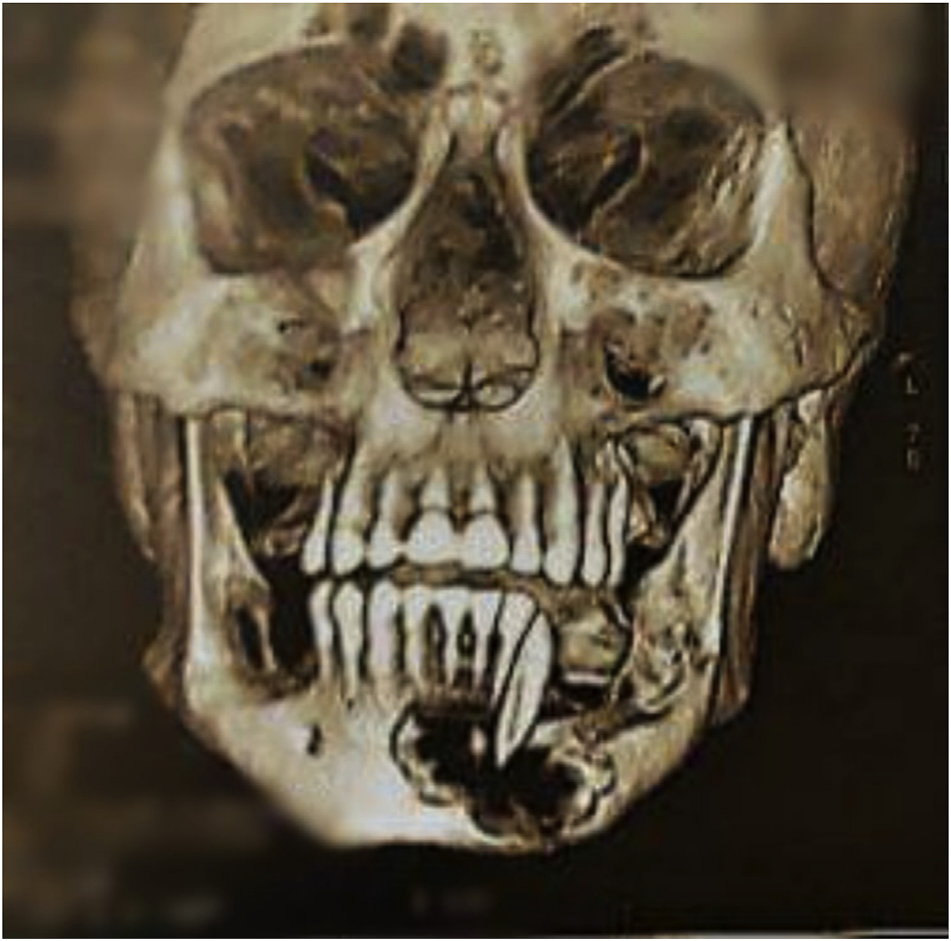
CT scan with 3D reconstruction: frontal incidence showing multiple radiolucent areas with well outlined borders on the mandible with a fracture and on the maxilla.

The presence of osteolytic lesions and mandibular fracture was confirmed by a subsequent 3D CT scan on multi plan reconstruction (MPR) and 3D images. (Fig. 2, Fig. 3, Fig. 4, Fig. 5, Fig. 6).

**The patient had teeth extractions of 1.4/2.5/2.6/2.7/3.3, an enucleation of the cystic lesions, and the osteosynthesis of the mandibular fracture using a miniplate.**

**Per operative exploration revealed a tissular aspect of both maxillary and mandibular cystic lesions.**

**Fig. 7:**
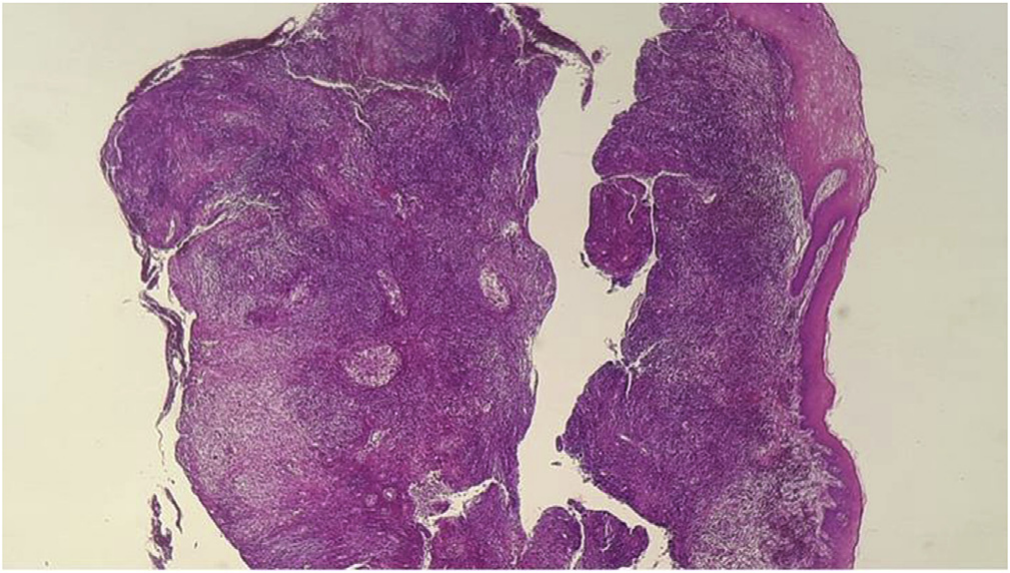
dense cellular infiltrate.

**Fig. 8:**
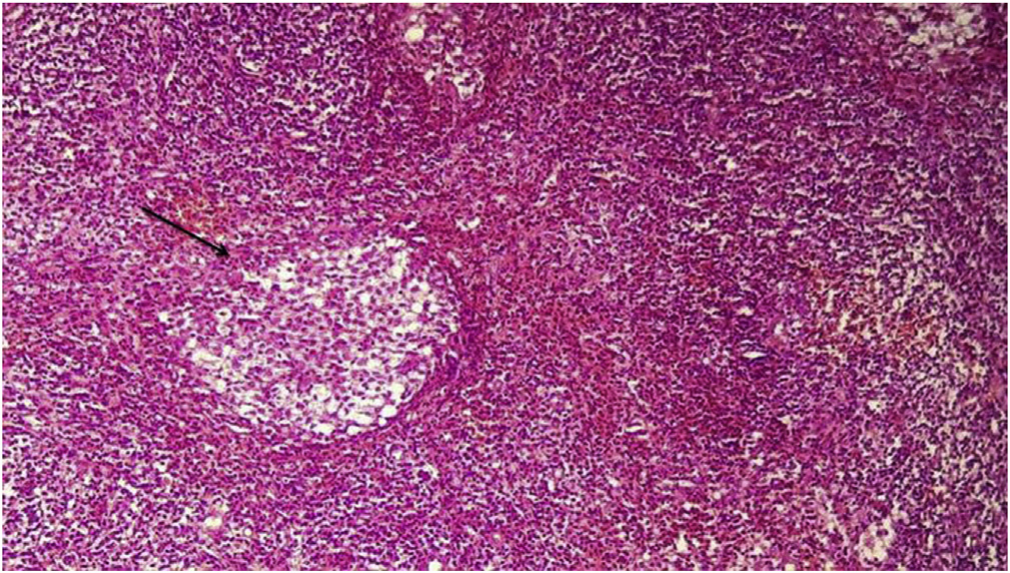
cells with abundant cytoplasmic content and perinuclear clearing assembled in nodules (arrow pointer).

**Fig. 9:**
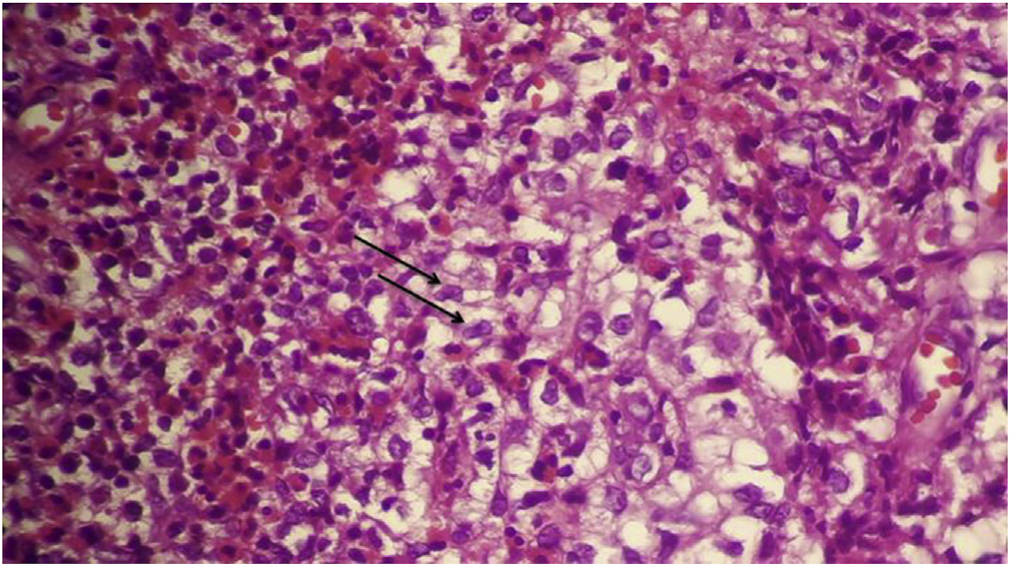
abundant eosinophilic cells with a kidney shaped nucleus (arrow pointer).

Histological and immunohistochemistry analysis of both lesions revealed a large cellular infiltrate (Fig. 7, Fig. 8, Fig. 9) attacking the epithelium surface with wide ulcerations. The inflammatory infiltrate mainly composed of mononuclear histiocytic cells was positive to CD1a.

According to these findings the final diagnosis of bifocal Langerhans cell histiocytosis of the oral cavity was made.

**Fig. 10:**
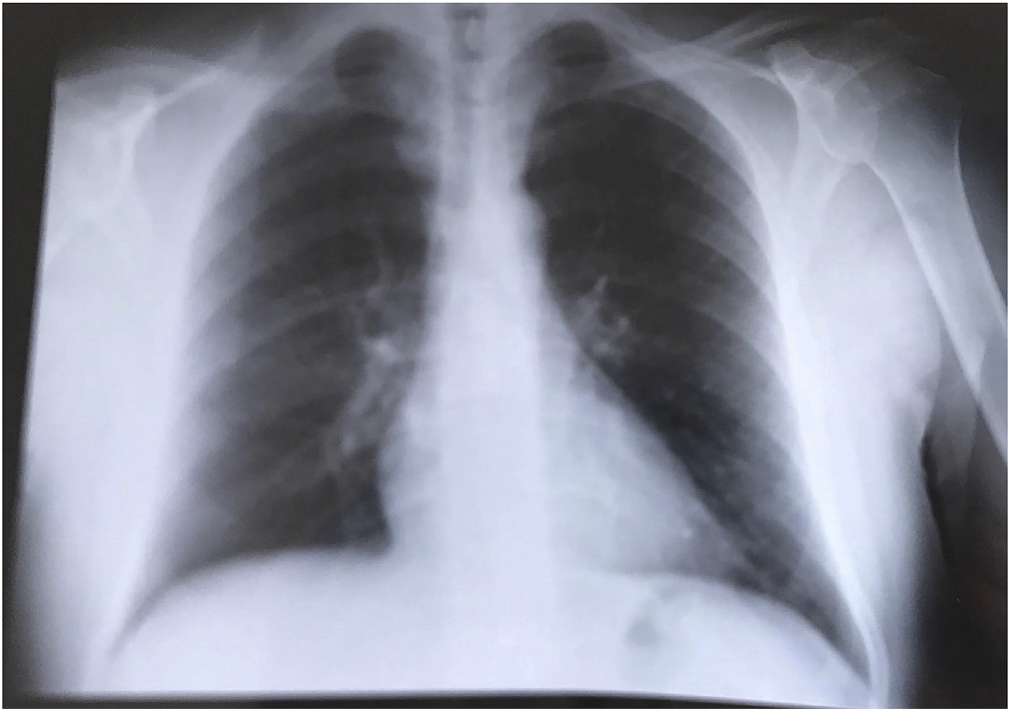
the chest X -ray showing no abnormalities.

Complete blood laboratory tests were all within normal limits, chest X-ray (Fig. 10) and radiographic examination of both femurs infirmed the presence of osteolytic lesions.

Ultrasonographic examination of the abdomen excluded involvement of “Risk Organs”(4).

The final diagnosis of single system LCH without involvement of “Risk Organs” was made.

**Fig. 11:**
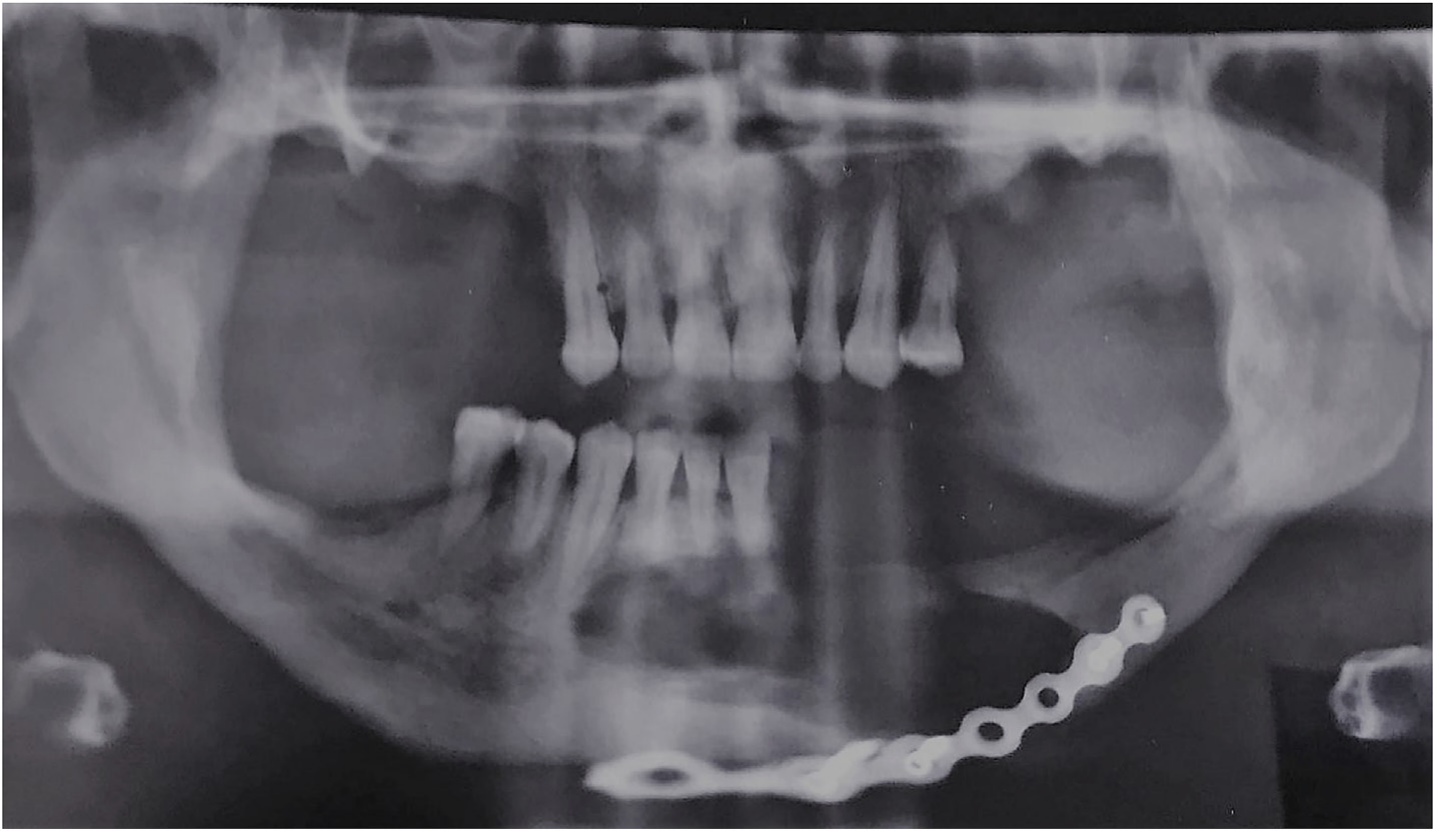
Post-operative orthopantomogram showing the good reduction of the mandibular fracture after bone osteosynthesis with a miniplate.

**The post-operative orthopantomogram was comforting showing a good fracture reduction of the mandibular pathologic fracture** (Fig. 11).

**Our patient clinical assessment respectively at 15 days post-operative and 1 month was comforting. However, the patient didn't come for his 6 month-control and couldn't be reached on the phone.**

## 3 Discussion

Langerhans cell histiocytosis is a rare disorder characterized by the proliferation of Langerhans dendritic cells. Incidence was estimated at 40 new cases per year in France and at 0,05 to 0,5cases/100000 children in the USA.

Mandibular location of histiocytosis can represent as much as 20,8% of non-odontogenic tumors [6]

It has variable clinical and radiological expressions which can vary from dental mobility to tumor development in the gingiva.

As far as we know, a maxillo-mandibular bifocal location is not frequent.The poorly known pathogenesis of LCH can include oral lesions as well as bone, mucosal and periodontal lesions(7).As for our case, the leading clinical symptom of LCH within mandibular and maxillary bones was pain which tend to be misdiagnosed as a marginal periodontal infection.

Mandibular bone lesions are the most common manifestation among facial lesions; the most frequent localizations are in an area between the cranium, the maxilla, and the mandible. Such lesions can be solitary intra-bony lesion and multiple alveolar lesions [7]

Periodontal lesions like gingival inflammation, ulceration, destruction of the keratinized gingiva, appear because of alveolar bone loss. Teeth surrounded by radiolucent defect related to LCH begin to move as “floating teeth” with consequent dental displacement and occlusion problems, pain and premature loss [[7], [8], [9], [10]].

**The aim of our clinical case report is to show that in front of a cystic lesion, and a radiolucent radiographic aspect of the alveolar ridge with integrity of oral mucosa, the diagnosis of histiocytosis should be raised**.

Consequently, a progressive “piano-key -like” third-degree teeth mobility in a young patient should be a wakeup call for the practitioner.

Thus histological proof is a key tool; its analysis showed the presence of Langerhans cells and a variable amount of eosinophils, neutrophils, mononuclear and polynuclear histiocytes and lymphocytes [11]

Certainly, an LCH lesion is not the first diagnosis that comes to mind in case of a bone osteolytic lesion or a pathologic fracture. Malignancies like primary bone tumors, metastases, multiple myeloma and even lymphoma or benign lesions like cystic tumors giant cell granuloma and osteomyelitis should be discussed first. It must be borne in mind that only with a correct integration of information regarding past medical history, an exhaustive physical examination, blood tests, radiographic exams and histological analysis can we reach towards the right diagnosis.

Treatment depends on the number and locations of the lesions. In case of a single system (SS) LCH, the treatment options may vary from a “wait and see” attitude to a non-aggressive surgery like for our patient with a possible local corticosteroid therapy or radiotherapy. The decision on the most appropriate approach should be based on clinical symptoms, the size and location of the lesion, and on any evidence of healing on imaging. Often, simple curettage during the diagnostic biopsy will result in healing, and further intervention may not be necessary.

Indications for additional treatment include unacceptable deformity, intense pain, and functional disability.

For lesions 2–5 cms in diameter, a biopsy and partial curettage is an option, like done for our patient. According to the clinical assessment no additional treatment was needed.

For multiple system locations (MS) LCH, systemic steroidal therapy, immunosuppressant agents, immune modulators or cytostatic drugs may be indicated [12,13]

The prognosis depends on various factors thus according to the report of the International Registry of the Histiocyte Society on adult LCH (IRHSA) which studied the clinical characteristics of 274 LCH cases from 13 nations, the survival at 5 years post diagnosis was 92.3% overall, 100% for patients with single-system disease, 87.8% for isolated pulmonary disease, and 91.7% for multisystem disease [12].

## 4 Conclusion

Facial locations of LCH are rare and even scarcer are those revealed by a pathologic mandibular fracture. This is to highlight the importance of investigating unusual findings such as a chronic pain or a third-degree teeth mobility in young patients with no history of periodontitis, performing radiograph examinations and histological analysis.

In this case a “piano-key-like” teeth mobility revealed a bifocal location of Langerhans cell histiocytosis.

Only the correct integration of the clinical, radiographic, histological, and biological data can allow the clinician to reach the final diagnosis. An early LCH diagnosis and a multidisciplinary treatment plan allows for the improvement of the patient ‘s prognosis and quality of life.

Written informed consent was obtained from the patient for publication of this case report and accompanying images.

## Provenance and peer review

Not commissioned, externally peer reviewed.

## Ethical approval

This type of study is exempt from ethnical approval in our institution.

## Sources of funding

None.

## Author contribution

Sabrine Maamouri: Writing the paper.

Marouen Ben Rejeb: Lecturing and correction of the paper.

## Registration of research studies

1. Name of the registry:
2. Unique identifying number or registration ID:
3. Hyperlink to your specific registration (must be publicly accessible and will be checked).

## Guarantor

Sabrine Maamouri.

## Declaration of competing interest

None.
